# Design of a comparative effectiveness evaluation of a culturally tailored versus standard community-based smoking cessation treatment program for LGBT smokers

**DOI:** 10.1186/2050-7283-2-12

**Published:** 2014-05-30

**Authors:** Alicia K Matthews, Elizabeth A McConnell, Chien-Ching Li, Maria C Vargas, Andrea King

**Affiliations:** University of Illinois at Chicago (UIC), Chicago, IL USA; DePaul University, Chicago, IL USA; Rush University, Chicago, IL USA; Howard Brown Health Center, Chicago, IL USA; University of Chicago, Chicago, IL USA; College of Nursing, University of Illinois at Chicago, 845 S. Damen Avenue, Chicago, IL 60612 USA

**Keywords:** Smoking cessation, LGBT, Culturally tailored

## Abstract

**Background:**

Smoking prevalence rates among the lesbian, gay, bisexual, and transgender (LGBT) population are significantly higher than the general population. However, there is limited research on smoking cessation treatments in this group, particularly on culturally targeted interventions. Moreover, there are few interventions that address culturally specific psychosocial variables (e.g., minority stress) that may influence outcomes. This paper describes the protocol for a comparative effectiveness trial testing an evidence-based smoking cessation program, Courage to Quit, against a culturally tailored version for LGBT smokers, and examines the role of culturally specific psychosocial variables on cessation outcomes.

**Methods/Design:**

To examine the effectiveness of a culturally targeted versus standard smoking cessation intervention, the study utilizes a 2-arm block, randomized, control trial (RCT) design. Adult LGBT participants (n = 400) are randomized to one of the two programs each consisting of a six-session group program delivered in a community center and an eight week supply of the transdermal nicotine patch. Four individualized telephone counseling sessions occur at weeks 2, 5, 7, and 9, at times of greatest risk for relapse. Study outcome measures are collected at baseline, and 1, 3, 6, and 12 months post quit date. Primary outcomes are expired air carbon monoxide verified 7-day point-prevalence quit rates at each measurement period. Secondary outcomes assess changes in cravings, withdrawal symptoms, smoking cessation self-efficacy, and treatment adherence. Additionally, study staff examines the role of culturally specific psychosocial variables on cessation outcomes using path analysis.

**Discussion:**

Determining the efficacy of culturally specific versus standard evidence based approaches to smoking cessation is a critical issue facing the field today. This study provides a model for the development and implementation of a culturally tailored smoking cessation intervention for LGBT participants and addresses a gap in the field by examining the role of culturally psychosocial variables associated with cessation outcomes.

**Trial registration:**

U.S. National Institutes of Health Clinical Trials NCT01633567 Registered 30 May 2012.

## Background

### Significance

In 2010, an estimated 25.2% of U.S. adults were current tobacco users, with 19.5% of adults reporting current cigarette smoking (King et al. 2012). However, the prevalence of smoking varies substantially by sexual orientation, with LGBT respondents significantly more likely to report cigarette smoking (32.8%) and cigar/cigarillo smoking (12.2%) than heterosexual respondents (19.5%; 6.5%) (King et al. 2012). Further, LGBT youth report higher past-30 day cigarette use (bisexual = 27.5% and lesbian/gay = 34.8%) compared with heterosexual youth (18.5%) (Rath et al. 2013). Although data are not currently available on cancer rates among LGBT smokers (Bowen and Boehmer 2007), morbidity and mortality due to tobacco use may be higher due to a greater prevalence of risk factors (e.g., heavy drinking and obesity) for diseases exacerbated by smoking (e.g., heart disease, diabetes, certain cancers, and HIV/AIDS) (Aaron et al. 2001). Despite both elevated smoking prevalence rates and increased vulnerability for smoking-related health disparities, few clinical trials of smoking cessation interventions for LGBT people exist (Hutchinson et al. 2006). Further, the Institute of Medicine report on LGBT health (Institute of Medicine of the National Academies 2011) failed to provide comprehensive goals to address this disparity. As such, LGBT smokers represent an important and underserved priority group for cessation efforts.

### Smoking cessation and LGBT populations

Research has shown that psychosocial variables related to smoking cessation may differ among subgroups (Fiore et al. 2008) and that considering cultural variation improves substance abuse treatment outcomes (Perez-Arce et al. 1993). A recent report by the Surgeon General highlights the need for additional research to determine whether tobacco dependence treatment programs have similar efficacy across diverse subgroups (U.S. Department of Health and Human Services 2000). Although research has identified differential cessation treatment needs of some groups (e.g., African Americans and women), we do not yet have precise data on the outcomes of LGBT smokers in mainstream smoking cessation interventions (Greenwood et al. 2005).

To date, there are few smoking cessation trials focused on LGBT smokers. The majority of the available studies are minimally tailored group based interventions delivered in community-based settings (Dickson-Spillmann et al. 2014; Eliason et al. 2012; Harding et al. 2004; Matthews et al. 2013a; Matthews et al. 2013b; Walls and Wisneski 2011). Although initial results are promising with end of treatment quit rates comparable to or better than those reported in the literature for smoking cessation programs in non-LGBT samples, there are concerns about the wide range in outcomes (16%-73% quit rates), small sample sizes, the absence of control groups, and the lack of objective verification of self-reported quit rates. Direct comparisons of interventions between LGBT and non-LGBT populations on non-tailored treatments are also limited. One intensive cessation intervention that was designed for the general population and combined bupropion, individual counseling, and nicotine replacement therapy found no significant difference in end of treatment quit rates for heterosexual and gay/bisexual male participants (57% vs. 58%, respectively) (Covey et al. 2009). As such, more extensive research is needed to determine the efficacy of smoking cessation interventions for LGBT populations.

### Consideration of LGBT related psychosocial factors

Compounding the modest intervention effects, there are large knowledge gaps regarding predicators of LGBT smoking cessation. In the general population, smoking cessation is strongly influenced by individually mediated predictors, including perceived benefits and barriers, self-efficacy, stage of readiness, and treatment adherence (Link et al. 2001; Spencer et al. 2002). Specific cultural factors (i.e., salience and identification with LGBT identity) are likely to play a role in smoking behaviors (Meyer 2003). Additionally, LGBT smokers are exposed to unique psychosocial stressors likely to influence smoking behaviors, such as elevated general stress (i.e., level of stress and number of stressful life events) and minority specific stress (i.e., internalized homophobia, sexual orientation concealment, discrimination events, stigma consciousness) (Meyer 2003; Steptoe et al. 1996; Rostosky et al. 2007). Minority stress (i.e., stress resulting from belonging to a stigmatized social category over and above general life stress; Meyer 2003) has been highlighted as an important but under-researched psychosocial influence on LGBT risk behaviors (Cochran and Mays 2000). As applied to LGBT individuals, minority stress is composed of five factors: (1) experiences of discrimination and violence, (2) stigma consciousness (awareness of discrimination), (3) sexual orientation concealment (level of ‘outness’), and (4) internalized homophobia (direction of societal negative attitudes toward the self) (Meyer, 2003). Presumed antecedents to minority stress (e.g., token minority status) have been used to explain mental and physical health outcomes in diverse samples (Clark et al. 1999). Further, minority stress for LGBT individuals has been associated with lower job satisfaction, increased distress and health related problems (Cochran and Mays 2000; Clark et al. 1999). However, we are not aware of any published studies that have used the minority stress framework to guide the development of a targeted risk reduction intervention. Given the strong and positive relationships between stress and/or negative affect and smoking (Steptoe et al. 1996), a minority stress model is a highly plausible heuristic for exploring predictors of smoking cessation outcomes in LGBT individuals. Thus, more research is needed to examine culturally specific individual mediators of cessation for LGBT smokers, including salience and identification with LGBT identity and community, general stress, and minority specific stress related to LGBT identity.

### The present study

**Figure 1:**
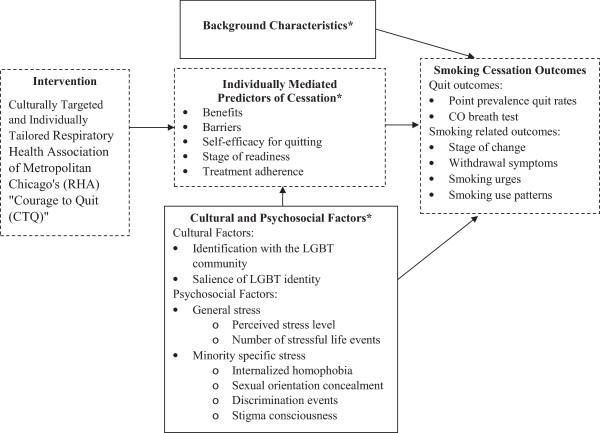
**Proposed smoking behavior change conceptual framework.** Note: *Intervened on in the CTQ intervention.

This study addresses an important gap in the literature by examining the effectiveness of a culturally targeted LGBT smoking cessation intervention in a randomized control trial. Additionally, we aim to examine the influence of LGBT specific cultural factors on smoking cessation. Our conceptual model is based on an integration of the core constructs of the Transtheoretical Model (TTM; Prochaska and Velicer 1998) and the Health Belief Model (HBM); (Becker et al. 1977) and emphasizes the multidimensional nature of health behavior change. In our model (see Figure 1), we posit behavior change is incremental in nature, shaped by health beliefs (as specified by the HBM), and associated with distinct stages (as in the TTM). In our intervention, we target individually mediated predictors of cessation at each stage of behavior change, including treatment adherence. This approach was used successfully in smoking cessation, mammography screening, nutritional intake, and exercise behavior (Campbell et al. 1994; Longshore and Grills 2000; Resnicow et al. 2001; Skinner et al. 1999; Campbell et al. 1994; Champion et al. 2003). The primary comparison in this study is between the culturally targeted (CTQ-CT) versus a standard Respiratory Health Association of Metropolitan Chicago’s (RHA) “Courage to Quit (CTQ)”, non-targeted program. However, our model also provides a framework to understand how the CTQ-CT affects smoking behavior, as well as the mediating or moderating factors that influence outcomes. Although most health behavior change models have been critiqued for not including a cultural component, our model incorporates psychosocial and cultural factors (e.g., minority stress) unique to the LGBT population along with generic predictors of cessation.

### Specific aims

The primary aim of this study is to examine the efficacy of culturally targeted CTQ-CT smoking cessation intervention compared to the standard CTQ program, in conjunction with nicotine replacement and peer support, for smoking cessation in LGBT smokers over time. Secondary aims are to determine the relationships of the cultural (i.e., identification with LGBT community and salience of sexual orientation identity) and psychosocial (i.e., indicators of general and minority stress) factors on smoking cessation outcomes using path analysis. Our primary hypothesis tests whether quit rates will be higher among individuals randomized to receive the CTQ-CT versus those who receive the standard CTQ program. We further hypothesize that higher levels of general and minority specific stress may be related to worse outcomes in smoking quit rates and these effects are moderated by treatment type.

## Methods

### Study design

**Figure 2:**
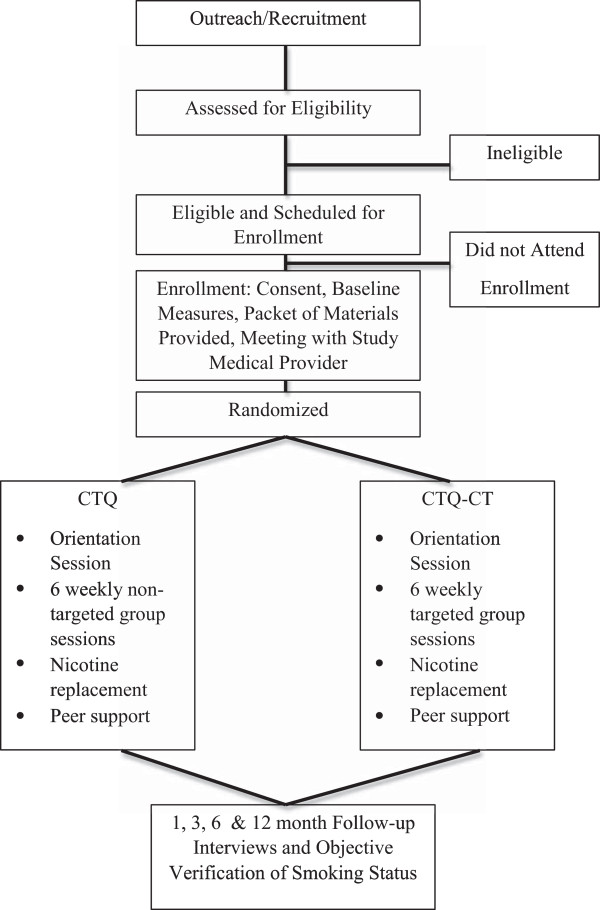
**Illustration of RCT study design and participant flowchart.**

A prospective 2-group randomized experimental design is proposed to test study hypothesizes related to the added benefit of the culturally tailored CTQ program (CTQ-CT) versus the standard CTQ program. The primary smoking cessation outcome examines point prevalence abstinence (i.e., no smoking, not even a puff, for previous 7 days). To determine short and longer-term cessation outcomes, self-report outcomes are objectively verified (using carbon monoxide [CO] testing) at 1 month, 3, 6, and 12 month follow-up time points. For an overview of the RCT study design, see Figure 2. Study activities have been reviewed and approved by the institutional review boards of The University of Illinois at Chicago (protocol identification number 2010–0538) and the Howard Brown Health Center (protocol identification number 10-163-100) community partner.

### Recruitment and retention

Participants will be 400 (200 CTQ-CT and 200 CTQ) LGBT tobacco smokers recruited from the community. This project is housed in the Behavioral Research Department of Howard Brown Health Center (HBHC), our community partner. Howard Brown Health Center is a nationally known LGBT health organization with a thirty-year history of conducting LGBT behavioral research. Recruitment takes place at HBHC through flyers about the study, information provided by clinic staff, and voluntarily provided email databases. Active community outreach consists of street outreach recruitment at street and venue locations, including bars, clubs, circuit parties, festivals, gyms, gay businesses, and other locations where LGBT socialize or congregate. Passive community outreach includes flyers, referrals, word of mouth, and posted advertisement on email listservs and websites, including social media sites. Participants are considered eligible if they: 1) self-identify as lesbian, gay, bisexual, or transgender, 2) are ages 18–65, 3) are current cigarette smokers (more than 5 packs in lifetime AND past year smoking AND 4 or more days per week AND CO air expired reading of ≥8 ppm), 4) express a desire to quit smoking (at least a 5 on a 10-point Likert scale), 5) agree to attend behavioral counseling sessions, be randomized, and be followed-up with, 6) agree to use nicotine patch and have no prior adverse reactions to patches, and 7) have a stable residence and telephone. Readiness and motivation to quit are key inclusion criteria. Research assistants offer self-help manuals to the less motivated (<5 on desire to quit smoking scale) and encourage them to attend the program at a more appropriate time.

Our goal is to achieve an 80% retention rate or better over the study period. Retention activities include providing incentives at each data collection point, providing a bonus incentive (lotteries) for those who complete all data collection points, providing giveaways such as pens with the study logo on them, sending birthday and holiday cards, and training staff on participant engagement and retention.

### Accrual and enrollment

A dedicated research phone line at the HBHC is used to enroll participants. Trained research assistants (RAs) answer calls, explain the study and assess eligibility for participation. The screening interview is a 10–15 minute semi-structured list of background characteristics, contact information, general medical condition, medications, and brief questions on smoking history. Eligible and interested individuals are given an appointment time at HBHC for an in-person screening with a study RA. At the first meeting, all participants sign informed consents, complete baseline measures, and receive an initial packet of study materials. Participants also meet with the study medical provider for a general health screening to evaluate any contraindications for nicotine replacement. Baseline measures are completed on a laptop computer in both an interviewer-administered and self-administered format.

### Randomization

Participants are randomized to CTQ-CT or non-targeted CTQ groups after eligibility and baseline screenings. The study statistician conducts the permuted-block randomization using a software program developed by programmers at UIC. This program uses random digits to assign code numbers in random blocks of equal control and experimental subject assignments. The study statistician place the results of the assignments in sealed, solid envelopes. All study participants are blinded and retain no knowledge of CTQ or CTQ-CT group assignment.

### Power analysis

Sample size and power for the study are based on detecting a 20% significant difference in cessation rates between the CTQ-CT and CTQ group in cross-sectional multinomial logistic regression models, in longitudinal survival analyses and in structural equation models. The logistic regression of smoking cessation on a continuous, normally distributed variable (e.g., perceived barriers) with a sample size of 340 observations achieves 85% power at 0.05 to detect the probability of change from the value of 0.05 at the mean of 0.16 when it is increased to one standard deviation above the mean. This change corresponds to an odds ratio of .40 (with an adjustment other independent variables explaining 20% of the variance) (Hsieh et al. 1998). Thus, approximately 400 subjects are required to provide a sample size of 340 participants for the survival analyses. Finally, in addition to sample size, the power for estimating and testing structural equation models depends on the numbers of free parameters and observed variables in each model (Saris and Satorra 1993). Sample sizes of 200–300 are generally regarded as effective in most cases for assessing model fit at an alpha of .05 (Boomsma 1982; Hoelter 1983; Schumacker and Lomax 1996); with 340 participants we will have adequate power to detect close-fitting models with 12 or more df, including tests of mediation (i.e., program and NRT adherence and treatment outcomes).

### Procedures

Participants are enrolled on a continuous basis. Behavioral counseling consists of six weekly 90-minute smoking cessation therapy sessions commencing two weeks before the quit date. Each session is preceded by a short interview with a research assistant to obtain self-report psychosocial and substance use information, obtain carbon monoxide measures, and distribute nicotine replacement. Nicotine replacement use begins on the quit date and continue for eight weeks. Post treatment follow-up interviews take place in person at 1, 3, 6, and 12 months after the quit date. These ½ -1 hour follow-up sessions with the research assistant include subjective questionnaires and objective measures (CO). For participants unable to complete interview in person at HBHC, 1, 3, 6, and 12 month follow-up assessments are completed remotely, over the phone. Research assistants collecting participant measures during follow-up interviews are blinded to treatment condition.

### Nicotine replacement

All participants are evaluated by the study medical provider to determine the appropriateness of nicotine replacement use (i.e., Nicoderm CQ: GlaxoSmithKline Consumer Healthcare, Pittsburgh, PA). At each visit, study staff distributes nicotine replacement therapy for each participant. An extra supply of three patches is given at each interval as back-ups if patches fall off, get misplaced, or if the subject does not attend the next session and needs an interim supply. Standard patch instructions are provided to each participant. Per manufacturer’s instructions for dosage, if a participant smokes 10 or more cigarettes per day, the initial dose is 21 mg for four weeks, 14 mg for two weeks, and then 7 mg for the final two weeks. Modifications of dosing level are adjusted, as needed, in conjunction with study medical provider in individuals who experience a substantial decline in smoking levels over the first two weeks in treatment (estimated at <10% of the sample) or who were lighter smokers at baseline.

### Intervention counselor training

A professional and a lay counselor who identify as LGBT facilitate each smoking cessation group. Both professional and lay counselors provide telephone support between sessions, as requested by participants. Process data (e.g., length and frequency of additional support contacts) is collected. The study primary investigator (A.M., clinical psychologist) takes primary responsibility of the training and supervision of counselors for the CTQ-CT and the CTQ program. To reduce contamination effects based on counselor training, group based supervision takes place on a monthly basis. Peer-support training is based on an established training model (Johnson et al. 2005). Former LGBT smokers serve as peer support staff (n = 6; Koehinger et al. 2005) and are trained in 4 weekly 60-minute sessions that cover: 1) cognitive/behavioral approaches used in the CTQ program, 2) smoking related health information, 3) motivational interviewing techniques, 4) strategies for eliciting and addressing barriers to smoking cessation for LGBT persons, 5) roles and responsibilities of peer support persons, 6) participant confidentiality, 7) helping participants deal with smoking triggers, cravings and lapses, 8) listening and feedback skills, and 9) providing non-judgmental support. Group leaders complete pre and post-measures to assess effectiveness of the peer support training. Ten percent of group sessions are audiotaped and reviewed by an independent rater for fidelity checks.

### Courage to quit smoking cessation treatment program

The Courage to Quit® (CTQ) program (developed by A.K.) was first implemented in 2008 by the Respiratory Health Association of Chicago (RHA) in partnership with community agencies with the goal of reaching underserved, minority, and low income smokers. The program is given at no- or low-cost (nominal fees for materials) and sessions are conducted in community health centers, non-profit organizations, faith-based organizations (e.g., churches), housing programs, hospitals, substance abuse treatment centers, and academic institutions. Group sizes average 10–20 participants. The first five sessions are held weekly. The third week is the target quit date, followed by the fourth (one week later), fifth (one week after session four), and the sixth (two weeks later) sessions. The program has been used as a platform treatment in clinical trials with six-month biochemically confirmed point-prevalence quit rates ranging from 19–35% (King et al. 2006, 2012; Matthews et al. 2009). The treatment modules include a progression of topics incorporating evidence-based behavioral, cognitive, and motivational smoking cessation strategies as outlined in the U.S. Public Health Service Clinical Practice Guidelines for Treating Tobacco Use and Dependence (Fiore et al. 2008).

Each session of the CTQ program has a particular emphasis. Group participation is encouraged in session one, which focuses on health benefits and relaxation techniques. Session two emphasizes motivation and covers nicotine addiction and peer support. Session three (quit week) provides testimonies of ex-smokers, offering hope that quitting is possible. Information on nicotine replacement and symptoms of recovery (withdrawal symptoms) is given, and participants commit to quit smoking for two days. Session four focuses on benefits of cessation and covers recovery symptoms and the grief process. Session five emphasizes beginning a new life as a nonsmoker: long-term goal setting, planning for social and stressful situations and weight management concerns are discussed. Session six introduces assertive communication tools and exercise as a long-term maintenance strategy. The final session recaps the program, discusses relapse prevention, and includes a group celebration of their new lifestyle. Group members who miss a session receive follow-up from the group facilitator before the next session. Individual or small-group make-up sessions are conducted so that all participants receive the full intervention content.

### Culturally tailored courage to quit program (CTQ-CT)

The five strategies outlined by Kreuter et al. (2002) are used to target the CTQ program to be culturally relevant to LGBT smokers: (1) peripheral strategies (e.g., culturally appropriate packaging, including images and exemplars with LGBT individuals); (2) evidential strategies (e.g., enhancing perceived relevance by presenting evidence of impact of smoking on LGBT); (3) linguistic strategies (e.g., using language relevant to the LGBT community); (4) constituent-involving strategies (e.g., including facilitators and peer support ‘buddies’ who are LGBT); and (5) sociocultural strategies (e.g., discussing smoking-related risks within the context of the broader social and cultural values of LGBT).

The control condition is the standard CTQ program. Professional and lay counselors are culturally sensitive. Control group participants complete the same measures and procedures as the CTQ-CT group members, including the follow-up schedule and peer support. Peer support telephone sessions take place at intervals of highest anxiety of anticipated quitting (week 2) and greatest risk for early relapse (weeks, 5, 7 and 9). (See Table 1 for overview of session topics).

**Table 1:** **Overview of the interventions**

	CTQ	CTQ-CT
**Orientation & Session 1**	Welcome participants, answer questions about the program, assess readiness to quit encourage group participation.	CTQ Orientation. Discussion of general and culturally specific LGBT determinants of smoking (e.g. social norms, uptake, tobacco industry targeting).
	Focus on group support. Discuss health benefits, describe models of nicotine addiction, focus on motivation for cessation, discuss nicotine replacement uses and function.	CTQ Session 1. Discussion of LGBT health, teach relaxation techniques
**Session 2**	Focus on factors leading to smoking, stress reduction management, encourage peer one-on-one support and social support.	CTQ Session 2. Discussion of study development phase outcomes, focus on LGBT specific factors of smoking uptake and triggers, discussion of additional support resources, quit day preparation.
**Session 3**	Quit week, nicotine replacement therapy (NRT), offer hope that quitting is possible, provide information on NRT, discuss symptoms of recovery (withdrawal), participants commit to a quit smoking attempt.	CTQ Session 3. Provide testimonies of ex-smokers, craving and withdrawal management.
**Session 4**	Focus on barriers and facilitators to cessation. Discuss recovery symptoms, relapse prevention, weight management concerns and the grief process.	CTQ Session 4. Discussion of LGBT related health and weight concerns, discussion of study development phase testimonies.
**Session 5**	Focus on goal setting and skill building. Discuss long-term goal setting, planning for social and stressful situations.	CTQ Session 5. Review of relapse causes and prevention, development of social support, and reminder of smoking goals.
**Session break**		
**Session 6**	Recap the program and review challenges of previous week. Focus on long-term maintenance. Introduce anger management tools, discuss relapse prevention, and celebrate new lifestyle.	CTQ Session 6. Review of study development phase testimonies.

### Measures

Table 2 shows the measures and time points for assessments. *Baseline measures* include demographics (including self-reported same and opposite-sex attraction; Diamond 2000), depressive symptoms (Beck et al. 1961), nicotine dependence (Heatherton et al. 1991), alcohol dependence (Selzer 1971), and expired-air carbon monoxide.

**Table 2:** **Summary of study measures**

Study variables	Assessment point	Measurement scale	Reference
**Smoking cessation outcomes: quit rates and smoking related outcomes**			
Biochemical verification of smoking status	All time points	Expired air CO breath test, α = .81, sensitivity of 78%, specificity of 91%	Smokerlyzer, Bedfort Corp., NJ
Point prevalence smoking quit rates	All time points	Time-Line Follow Back (TLFB) interview, α = .80	Sobell and Sobel 1995; Sobell et al. 1979
Smoking use patterns	All time points	Time-Line Follow Back (TLFB) interview, α = .80	Sobell and Sobel 1995; Sobell et al. 1979
Withdrawal symptoms	All time points	Minnesota Nicotine Withdrawal Scale, 8-items, α = .80-.85	Hughes and Hatsukami 1986
Smoking urges	All time points	Brief Questionnaire of Smoking Urges, 10-items, α = .89	Cox et al. 2001; Tiffany and Drobes, 1991
**Individually mediated predictors of cessation**			
Perceived benefits	Baseline, 3 and 6 mo.	Perceived positive outcomes associated with quitting, α = .70.	Menon et al. 2003
Perceived barriers	Baseline, 3 and 6 mo.	Obstacles that inhibit smoking cessation, α = .70.	Menon et al. 2003
Self-efficacy for quitting	Baseline, 3 and 6 mo.	Smoking Abstinence Self-efficacy Scale, 4-items, α = 85-.89	Tiffany and Drobes 1991
Stage of change	Baseline, follow-ups	Smoking Cessation Contemplation Ladder, 0–10 measure	Biener and Abrams 1991
**Psychosocial factors**			
Perceived stress	Baseline, 3 and 6 mo.	Perceived Stress Scale, 14-items	Cohen et al. 1983; Cohen and Williamson 1988
# of stressful life events	Baseline, 3 and 6 mo.	The Scaling of Life Events Questionnaire, 61-items	Paykel et al. 1971
**Minority stress factors**			
Internalized homophobia	Baseline	Internalized Homophobia Scale, 9-items, α = .79	Herek et al. 1997
Sexual orientation concealment	Baseline	Level of Outness Scale, 3 items, α = .86	Bowen et al. 2006
Experience of discrimination	Baseline	Experiences of Discrimination Scale [EDS], 45 items, α = .74.	Krieger et al. 2005
Stigma consciousness	Baseline	Modified Devaluation-Discrimination scale, 12-items, α = .88	Link 1987; Link et al. 2001
**Cultural factors**			
Cultural identification	Baseline	Level of involvement with the LGBT community, 10-items, α = .76	Bowen et al. 2006
Cultural salience	Baseline	To be developed for this study	---
**Background characteristics**			
Demographics	Baseline	Age, education, gender, income, insurance, sexual orientation, marital status and race	---
Depression	Baseline	Beck Depression Inventory, 20-items, α = .86	Beck et al. 1961
Nicotine dependency	Baseline	Fagerstrom Test for Nicotine Dependence, 6-items, α = .61	Heatherton et al. 1991
Illicit drug use	Baseline	Measures of illicit drug use	-----
Alcohol use	All time points	Time Ling Follow Back, α = .76-.97, Michigan Alcohol Screening	Sobell and Sobel 1995; Sobell et al. 1979; Hughes et al. 2003

Note: All Time Points = Baseline, Weekly during intervention, and 1, 3, 6, 12 month follow-up interviews.

*Smoking cessation outcomes* are measured using multiple definitions of quit rates (Hughes et al. 2003; Hughes and Hatsukami 1986): point prevalence smoking quit rates (i.e., no smoking during the past seven days; derived from Time Line Follow-Back interviews; Sobell and Sobel 1995; Sobell et al. 1979); continuous abstinence (i.e., abstinence period that began on quit date); prolonged abstinence (i.e., no smoking in the past 30 days); and biochemical verification of smoking status using expired-air carbon monoxide at each study visit and follow-up session (Smokerlyzer, Bedfont Corp., Medford, NJ). Additional smoking cessation outcomes include: withdrawal symptoms (Hughes and Hatsukami, 1986); urges to smoke (Cox et al. 2001); and temporal measures of consumption and fluctuations in use patterns over time for cigarettes and alcohol (derived from the Modified Time-Line Follow Back Interview; Sobell and Sobel 1995; Sobell et al. 1979). This range of outcomes may provide clues as to the mechanism of action of targeted interventions on smoking (Hughes et al. 2003).

*Individually mediated predictors of cessation* are be assessed using: perceived benefits, perceived barriers, and self-efficacy of quitting (Health Belief Model Constructs, Becker et al. 1977; Smoking Abstinence Self-Efficacy Scale, Velicer et al. 1990); stage of readiness for smoking cessation (Prochaska and Velicer 1998); extent of considering quitting (Biener and Abrams 1991); adherence to the nicotine replacement therapy and side effects (derived from the Timeline follow-up interviews, daily participant logs, and weekly patch counts; Ahluwalia et al. 1998).

Study participants complete a number of *cultural and psychosocial variables*. First, level of identification with the LGBT community (Bowen et al. 2006) is assessed. Second, minority specific stress is measured using: internalized homophobia (Herek et al. 1997); stigma consciousness (adapted from Link 1987; Link et al. 2001); experience of discrimination and sexual-minority related stressors (Krieger et al. 2005); and sexual orientation concealment (Bowen et al. 2006). Third, psychosocial stress is assessed using both perceived stress (Cohen et al. 1983; Cohen and Williamson 1988) and scaling of life events (Paykel et al. 1971).

### Analytic plan

To test the effectiveness of the CTQ-CT program compared to the CTQ standard program, we will fit a series of regression models to determine independent predictors of cessation (i.e., point prevalence, continuous abstinence, and prolonged abstinence) and to test the independent effect of treatment condition at each wave of data collection. To address the question of efficacy over time, we aim to conduct survival analyses (i.e., discrete-time and continuous-time hazard models) to estimate hazard functions (i.e., risk of relapse) and survivor functions (i.e., probability of sustained quitting), identify periods when relapse is likely, and determine the shape of the hazard function (whether the risk increases, decreases or remains constant over the year of participation). To test both hypotheses, we will first estimate correlations among all measures of cultural and psychosocial processes, psychosocial indicators of general stress, and minority specific stressors. We will then fit covariance structure models to the data to estimate the simultaneous relationships among the variables and to test for both mediator and/or moderator effects. Analyses for this aim are conceptually depicted in Figure 1. In examining these mechanisms we will employ established procedures for the construction and assessment of product terms in SEM (Jaccard and Wan 1996; Jöreskog and Yang 1996). In addition, model fit will be compared across subgroups (e.g., lesbians, bisexuals, gay men, and transgendered participants) via multiple group SEMs and formal testing of parameter equivalence using nested difference-of- χ^2^ tests (Byrne 1998; Hayduk 1987).

## Discussion

This is the first randomized control trial to test the efficacy of a group based and culturally tailored smoking cessation intervention for LGBT participants. This trial provides a number of significant contributions. First, the CTQ-CT program builds on an established smoking cessation intervention (CTQ) by incorporating a number of cultural tailoring strategies (i.e., Kreuter et al. 2002). Second, our approach is innovative in that the analytical model takes into account both general and culturally specific individually mediated predictors of smoking cessation. This addresses a gap in the literature, as most models fail to take into account or assess the role of cultural factors as predictors to account for within-group variability. Third, the strong methodological design of this study allows us to isolate the effect of treatment group while controlling for a number of other factors. Fourth, we use biochemical verification of smoking status (i.e., CO monitoring) in addition to self-report measures. Fifth, we follow up with study participants over a period of 12 months to determine the effect of the CTQ and CTQ-CT interventions over time.

While this study has a number of strengths and contributes to existing gaps in the literature, we also acknowledge several limitations. The sample is drawn from Chicago, and additional research should be conducted with LGBT communities in other geographic areas. Only participants highly motivated to quit and smoking 4 or more days per week with CO air expired reading of ≥8 are included in the study. This sample may differ from participants who are less motivated to quit or who smoke less frequently.

Overall, this research addresses a number of gaps in the existing literature on culturally targeted smoking cessation interventions and contributes a culturally-informed model of cessation with a rigorous methodological design. Findings have the potential to shape knowledge and future research about health behavior change for specific populations and reduce smoking-related health disparities for LGBT communities.

## References

[CR1] Aaron DJ, Markovic N, Danielson ME, Honnold JA, Janosky JE, Schmidt NJ (2001). Behavioral risk factors for disease and preventive health practices among lesbians. American Journal of Public Health.

[CR2] Ahluwalia JS, McNagny SE, Clark WS (1998). Smoking cessation among inner‒city African Americans using the nicotine transdermal patch. Journal of General Internal Medicine.

[CR3] Beck AT, Ward CH, Menderson M, Mack J, Erbaugh J (1961). An inventory for measuring depression. Archives of General Psychiatry.

[CR4] Becker MH, Maiman LA, Kirscht JP, Haefner DP, Drachman RH (1977). The health belief model and prediction of dietary compliance: a field experiment. Journal of Health and Social Behavior.

[CR5] Biener L, Abrams D (1991). The contemplation ladder: validation of a measure of readiness to consider smoking cessation. Health Psychology.

[CR6] Boomsma A, Joreskog KG, Wold H (1982). The robustness of LISREL against small sample sizes in factor analysis models. Systems Under Indirect Observation. Part I.

[CR7] Bowen DJ, Boehmer U (2007). The lack of cancer surveillance data on sexual minorities and strategies for change. Cancer Causes & Control.

[CR8] Bowen DJ, Powers D, Greenlee H (2006). Effects of breast cancer risk counseling for sexual minority women. Health Care for Women International.

[CR9] Byrne BM (1998). Structural Equation Modeling with LISREL, PRELIS, and SIMPLIS.

[CR10] Campbell MK, DeVellis BM, Strecher VJ, Ammerman AS, DeVilles RF, Sandeler RS (1994). Improving dietary behavior: the effectiveness of tailored messages in primary care settings. American Journal of Public Health.

[CR11] Champion VL, Maraj MS, Hui S, Perkin A, Tierney W, Menon W, Skinner CS (2003). Comparison of tailored interventions to increase mammography screening in nonadherent older women. Preventive Medicine.

[CR12] Clark R, Anderson NB, Clark VR, Williams DR (1999). Racism as a stress for African Americans: a biopsychosocial model. American Psychologist.

[CR13] Cochran S, Mays V (2000). Relation between psychiatric syndromes and behaviorally defined sexual orientation in a sample of the US population. American Journal of Epidemiology.

[CR14] Cohen S, Williamson GM, Oskamp S (1988). Perceived stress in a probability sample of the United States. S Spacapan, S Oskamp (Eds), The social psychology of health.

[CR15] Cohen S, Kamarck T, Mermelstein R (1983). A global measure of perceived stress. Journal of Health and Social Behavior.

[CR16] Covey LS, Weissman J, LoDuca C, Duan N (2009). A comparison of abstinence outcomes among gay/bisexual and heterosexual male smokers in an intensive, non-tailored smoking cessation study. Nicotine & Tobacco Research.

[CR17] Cox LS, Tiffany ST, Christen AG (2001). Evaluation of the brief questionnaire of smoking urges (QSU-brief) in laboratory and clinical settings. Nicotine & Tobacco Research.

[CR18] Diamond LM (2000). Sexual identity, attractions, and behavior among young sexual minority women over a 2-year period. Developmental Psychology.

[CR19] Dickson-Spillmann M, Sullivan R, Zahno B, Schaub MP (2014). Queer quit: a pilot study of a smoking cessation programme tailored to gay men. BMC Public Health.

[CR20] Eliason MJ, Dibble SL, Gordon R, Soliz GB (2012). The last drag: an evaluation of an LGBT-specific smoking intervention. Journal of Homosexuality.

[CR21] Fiore MC, Jaen CR, Baker TB, Bailey WC, Benowitz N, Curry SJ (2008). Treating tobacco use and dependence: 2008 update US public health service clinical practice guideline executive summary. Respiratory Care.

[CR22] Greenwood GL, Paul JP, Pollack LM, Binson D, Catania JA, Chang J, Humfleet G, Stall R (2005). Tobacco use and cessation among a household-based sample of US urban men who have sex with men. American Journal of Public Health.

[CR23] Harding R, Bensley J, Corrigan N (2004). Targeting smoking cessation to high prevalence communities: outcomes from a pilot intervention for gay men. BMC Public Health.

[CR24] Hayduk LA (1987). Structural equation modeling with LISREL: Essentials and advances.

[CR25] Heatherton TF, Kozlowshi LT, Frecker RC, Fagerström K (1991). The Fagerström test for nicotine dependence: a revision of the Fagerström tolerance questionnaire. British Journal of Addiction.

[CR26] Herek GM, Cogan JC, Gillis JR, Glunt EK (1997). Correlates of internalized homophobia in a community sample of lesbians and gay men. Journal of the Gay and Lesbian Medical Association.

[CR27] Hoelter JW (1983). The analysis of covariance structures: goodness-of-fit indices. Sociological Methods & Research.

[CR28] Hsieh FY, Block DA, Larsen MD (1998). A simple method of sample size calculation for linear and logistic regression. Statistics in Medicine.

[CR29] Hughes JR, Hatsukami D (1986). Signs and symptoms of tobacco withdrawal. Archives of General Psychiatry.

[CR30] Hughes JR, Keely JP, Niaura RS, Ossip-Klein DJ, Richmond RL, Swan GE (2003). Measures of abstinence in clinical trials: issues and recommendations. Nicotine and Tobacco Research.

[CR31] Hutchinson MK, Thompson AC, Cederbaum JA (2006). Multisystem factors contributing to disparities in preventive health care among lesbian women. Journal of Obstetric, Gynecologic, & Neonatal Nursing.

[CR32] Institute of Medicine of the National Academies (2011). The health of lesbian, gay, bisexual, and transgender people: Building a foundation for better understanding.

[CR33] Jaccard J, Wan CK (1996). Lisrel approaches to interaction effects in multiple regression.

[CR34] Johnson RE, Green BL, Anderson-Lewis C, Wynn TA (2005). Community health advisors as research partners: an evaluation of the training and activities. Family Community Health.

[CR35] Jöreskog K, Yang F, Marcoulides G, Schumacker R (1996). Non-linear structural equation models: The Kenny-Judd model with interaction effects. Advanced Structural Equation Modeling.

[CR36] King AC, de Wit H, Riley RC, Cao D, Niaura R, Hatsukami D (2006). Efficacy of naltrexone in smoking cessation: a preliminary study and an examination of sex differences. Nicotine & Tobacco Research.

[CR37] King BA, Dube SR, Tynan MA (2012). Current tobacco use among adults in the United States: findings from the national adult tobacco survey. American Journal of Public Health.

[CR38] Koehinger S, Powers C, Masini B, McKirnan D, Cook S (2005). Annual Meeting of the American Public Health Association. Examining the effectiveness of a culturally tailored cessation program for LGBT smokers.

[CR39] Kreuter MW, Lukwago SN, Bucholtz DC, Clark EM, Sanders-Thompson V (2002). Achieving cultural appropriateness in health promotion programs: targeted and tailored approaches. Health Education and Behavior.

[CR40] Krieger N, Smith K, Naishadham D, Hartman C, Barbeau EM (2005). Experiences of discrimination: validity and reliability of a self-report measure for population health research on racism and health. Social Science and Medicine.

[CR41] Link BG (1987). Understanding labeling effects in the area of mental disorders: an assessment of the effects of expectations of rejection. American Sociological Review.

[CR42] Link BG, Struening EL, Neese-Todd S, Asmussen S, Phelan JC (2001). Stigma as a barrier to recovery: the consequences of stigma for the self-esteem of people with mental illnesses. Psychiatric Services.

[CR43] Longshore D, Grills C (2000). Motivating illegal drug use recovery: evidence for a culturally congruent intervention. Journal of Black Psychology.

[CR44] Matthews AK, Sánchez-Johnsen L, King A (2009). Development of a culturally targeted smoking cessation intervention for African American smokers. Journal of Community Health.

[CR45] Matthews AK, Conrad M, Kuhns L, Vargas M, King AC (2013). Project exhale: preliminary evaluation of a tailored smoking cessation treatment for HIV + African American smokers. AIDS Patient Care and STDS.

[CR46] Matthews AK, Li CC, Kuhns LM, Tasker TB, Cesario JA (2013). Results from a community-based smoking cessation treatment program for LGBT smokers. Journal of Environmental and Public Health.

[CR47] Menon U, Champion VL, Larkin GN, Zollinger TW, Gerde P, Vernon SW (2003). Beliefs associated with fecal occult blood test and colonoscopy use at a work-site colon cancer screening program. Journal of Occupational and Environmental Medicine.

[CR48] Meyer IH (2003). Prejudice, social stress and mental health in lesbian, gay, and bisexual populations: conceptual issues and research evidence. Psychological Bulletin.

[CR49] Paykel ES, Prusoff BA, Uhlenhuth E (1971). Scaling of life events. Archives of General Psychiatry.

[CR50] Perez-Arce P, Carr KD, Sorenson J (1993). Cultural issues in an outpatient program for stimulant abusers. Journal of Psychoactive Drugs.

[CR51] Prochaska JO, Velicer WF (1998). The transtheoretical model of health behavior change. American Journal of Health Promotion.

[CR52] Rath JM, Villante AC, Rubenstein RA, Vallone DM (2013). Tobacco use by sexual identity among young adults in the United States. Nicotine & Tobacco Research.

[CR53] Resnicow K, Jackson A, Wang T, De AK, McCarty F, Dudley WN, Baranowski T (2001). A motivational interviewing intervention to increase fruit and vegetable intake through black churches: results of the eat for life trial. American Journal of Public Health.

[CR54] Rostosky SS, Riggle E, Gray B, Hatton RL (2007). Minority stress experiences in committed same-sex couple relationships. Professional Psychology: Research and Practice.

[CR55] Saris WE, Satorra A, Bollen KA, Long JS (1993). Power evaluations in structural Equation Models. Testing Structural Equation Models.

[CR56] Schumacker RE, Lomax RG (1996). A Beginner’s Guide to Structural Equation Modeling.

[CR57] Selzer ML (1971). The Michigan alcohol screening test: the quest for a new diagnostic instrument. American Journal of Psychiatry.

[CR58] Skinner CS, Campbell MK, Rimer BK, Curry S, Prochaska JO (1999). How effective is tailored print communication?. Annals of Behavioral Medicine.

[CR59] Sobell LC, Sobel MB (1995). Alcohol timeline follow-back users’ manual.

[CR60] Sobell LC, Maisto SA, Sobel MB, Cooper AM (1979). Reliability of alcohol abusers’ self-reports of drinking behavior. Behavioral Research & Therapy.

[CR61] Spencer L, Pagell F, Hallion ME, Adams TB (2002). Applying the transtheoretical model to tobacco cessation and prevention: a review of the literature. American Journal of Health Promotion.

[CR62] Steptoe A, Wardle J, Pollard TM, Cannan L, Davies GJ (1996). Stress, social support and health-related behavior: a study of smoking, alcohol consumption and physical exercise. Journal of Psychosomatic Research.

[CR63] Tiffany ST, Drobes DJ (1991). The development and initial validation of a questionnaire on smoking urges. British Journal of Addiction.

[CR64] U.S. Department of Health and Human Services (2000). Reducing Tobacco Use: A report of the Surgeon General.

[CR65] Velicer WF, Diclemente CC, Rossi JS, Prochaska JO (1990). Relapse situations and self-efficacy: an integrative model. Addictive Behaviors.

[CR66] Walls NE, Wisneski H (2011). Evaluation of smoking cessation classes for the lesbian, gay, bisexual, and transgender community. Journal of Social Service Research.

[CR67] The pre-publication history for this paper can be accessed here: http://www.biomedcentral.com/2050-7283/2/12/prepub

